# Editorial: Design of Functional Nanostructured Polymer Materials: Synthesis, Characterization and Applications

**DOI:** 10.3389/fchem.2022.923054

**Published:** 2022-05-13

**Authors:** Vera Alejandra Álvarez, Guillermo Javier Copello, Roberto Fernández de Luis, Antonia Infantes Molina, Juan Manuel Lázaro-Martínez

**Affiliations:** ^1^ Consejo Nacional de Investigaciones Científicas y Técnicas (CONICET), Instituto de Investigaciones en Ciencia y Tecnología de Materiales (INTEMA), Grupo de Materiales Compuestos Termoplásticos (CoMP), Facultad de Ingeniería, Universidad Nacional de Mar del Plata, Mar del Plata, Argentina; ^2^ Universidad de Buenos Aires, Facultad de Farmacia y Bioquímica, Departamento de Ciencias Químicas, Buenos Aires, Argentina; ^3^ CONICET - Universidad de Buenos Aires, Instituto de Química y Metabolismo del Fármaco (IQUIMEFA), Buenos Aires, Argentina; ^4^ BCMaterials, Basque Center for Materials, Applications and Nanostructures UPV/EHU Science Park, Leioa, Spain; ^5^ Departamento de Química Inorgánica, Facultad de Ciencias, Universidad de Málaga, Málaga, Spain

**Keywords:** nanocomposite, grafting, crosslinking, characterization techniques, nanoscience

Modern problems sometimes require innovative solutions. Like many science fields, traditional polymer science would have reached an innovative plateau if it had not reinvented itself by the aid of modern technologies. Nowadays, polymers have exceeded their role as structural materials. In this sense, smart polymers, functional polymers, nanostructured polymers allow the development of materials and formulations with outstanding performances. These developments have been possible due to the interest of academic and industry researchers that have gathered through the years enough knowledge to manipulate, modify, characterize, and apply these polymers in a way that was unthinkable a decade ago. In this matter, nanotechnology has played a major role. The rise of nanotechnology at the beginning of this century has boosted the interest of its application as a panacea for any technological drawback.

As time goes by, nanotechnology has matured and the resultant developments can be found in the scientific literature as well as in patents and also in commercial products. This Research Topic gathers a portion of these developments. It revisits the relevance of nanotechnology in polymer science at different levels, from synthesis, characterization and applications of these materials.

Regarding synthesis, several strategies that confer a nanostructure to a material are reported in this Topic. Li et al. have made a nanostructured chitosan aerogel by lyophilization (Li et al.). Xiao et al. also achieved a nanostructured material whose internal morphology was endowed by the polymers used in the hydrogel assembly (Xiao et al.). They have observed the typical coral-like and dendritic 3D network structure of polyaniline (PANI). Also, the internal morphology of the material could be influenced by the amount of gelatin in the composite.

In a different approach, using nanostructures as molding agents, Thimmaiah et al. have developed a nanocomposite film by embedding lithium silver oxide (LAO) nanoparticles (NPs) in sodium alginate (Giriyappa Thimmaiah et al.). The authors highlight the relevance of avoiding phase separation in the system in order to achieve a homogeneous material. In this matter one can say that characterization is an important issue, and will predict or explain the material properties and operative behavior. In fact, Zhao et al. have developed a method to calculate the fractal dimension of non-ordered nano-polymeric microspheres (Zhao et al.). Interestingly, they have found that the calculated fractal dimension of polymeric microspheres was linearly correlated to the average particle size during the hydration of the sphere. The use of freely available and powerful software such as NIH ImageJ was indispensable for this work.

There is no doubt that when it comes to nanostructured materials, microscopies play a major role in the characterization. Nevertheless, all works in this Topic demonstrate that more statistically weighted methods are needed to characterize NPs, such as Dynamic Light Scattering (DLS), and that other specific techniques should be implemented in order to gain a deep insight in the material properties, such as the crystalline phases or crystallite size of the NPs, that can be studied by X-ray diffraction (XRD), or the polymer’s functional groups and interaction among, that can be analyzed by FT-IR.

The works from this Research Topic also demonstrate that applicability is one of the main goals for academic and industry researchers working in polymer chemistry. Li et al. and Xiao et al. have focused their works in the medicine field (Li et al.; Xiao et al.). The first authors have used the chemical nature and the nanostructure of the material as tools to control the release of ibuprofen. They have studied the release and permeation of the drug by means of medium pH and loading. The latter authors have developed a conductive antibacterial nanocomposite by mixing Ag NPs in a PANI/polyvinyl alcohol/gelatin matrix. For this application, together with the antimicrobial properties, the authors highlighted the relevance of testing the biocompatibility of the material.

Seeking biocompatibility, Thimmaiah et al. have chosen sodium alginate as the support matrix for LAO NPs for the development of portable electronics (Giriyappa Thimmaiah et al.). In this case, the dielectric nature of alginate hinders the conductivity transmission of LAO NPs. Therefore, the loading of the NPs and the electric parameters needed for the proper performance of the material needed to be studied.

In brief, this Research Topic, compiles novel developments regarding nanostructured polymer materials, by assessing new synthetic methods, their characterization and the study of the plausibility of their application in order to allow the readers from industry and academia to reach for new ideas for future progress.

